# The Proof-of-the Concept of Biochar Floating Cover Influence on Swine Manure pH: Implications for Mitigation of Gaseous Emissions From Area Sources

**DOI:** 10.3389/fchem.2020.00656

**Published:** 2020-08-07

**Authors:** Zhanibek Meiirkhanuly, Jacek A. Koziel, Andrzej Bialowiec, Chumki Banik, Robert C. Brown

**Affiliations:** ^1^Department of Agricultural and Biosystems Engineering, Iowa State University, Ames, IA, United States; ^2^Faculty of Life Sciences and Technology, Wrocław University of Environmental and Life Sciences, Wrocław, Poland; ^3^Bioeconomy Institute and Department of Mechanical Engineering, Iowa State University, Ames, IA, United States

**Keywords:** area sources, emissions control, biochar, swine manure, spatial and temporal pH, buffer capacity, mass transfer, liquid-air interface

## Abstract

Mitigation of potentially hazardous and malodor compounds emitted from animal waste is needed to improve the sustainability of livestock agriculture. Bacteria control the generation of these compounds and also depend on the pH of manure. Influencing swine manure pH, especially on the liquid-air interface, may lead to a reduction of emission of odorous and hazardous compounds. The objective of this experiment was to test highly alkaline and porous (HAP) modified biochar with pH = 9.2 and red oak (RO) biochar with pH = 7.5 influence on swine manure pH acquired from the outdoor storage and deep pit storage under a barn. HAP and RO biochars were topically applied on the outdoor-stored (pH = 7.55), and pit (pH = 8.00) manures and spatial pH (every 1 mm of depth) were measured on days 0, 2, and 4. Results showed that HAP biochar increased outdoor-stored manure pH on day 4, particularly within the top 10 mm of depth, where pH ranged from 7.79 to 8.90, while in the case of RO pH ranged between 7.46 and 7.66, i.e., similar to control (7.57–7.64). Both biochars decreased pit-stored manure pH within the top 10 mm of depth (in comparison with the control pH of 8.36–8.47) to 8.19–8.30 (HAP), and 8.18–8.29 (RO) on day 4. However, differences were not considerable. The reason for the insignificant effect of biochars on pit manure was likely due to its higher buffer capacity in comparison with the outdoor-stored manure.

## Introduction

The increase in livestock production leads to an increase in the volume of manure storage and challenges to its utilization. Manure storage in open lagoons and outdoor storages can be a source of malodor and elevated concentrations of gases such as ammonia (NH_3_) (Grant and Boehm, 2018; Reyes et al., 2019), hydrogen sulfide (H_2_S) (Rumsey and Aneja, 2014), and greenhouse gases including methane (CH_4_), carbon dioxide (CO_2_), and nitrous oxide (N_2_O) (Hitaj et al., 2019). Moreover, volatile organic compounds (VOCs) (including sulfur-containing compounds, fatty acids, and phenolics) (Trabue et al., 2019) are also responsible for malodor from stored manure.

Zahn et al. (2001) reported the emission rate from the deep-pit barn of 2.38 g NH_3_ m^−2^ h^−1^ for ammonia, while hydrogen sulfide had an emission rate of 13.3 mg H_2_S m^−2^ h^−1^. According to the Environmental Protection Agency (EPA), 15% of greenhouse emissions are associated with manure management in the agricultural sector in the United States (The United States Environmental Protection Agency, 2020).

Solving the environmental problems related to livestock is a challenge for farmers, public, and regulatory agencies. A comprehensive solution that includes not only effectiveness but also practicality and low-cost is in high demand. Iowa State University Extension and Outreach Air Management Practices Assessment Tool summarized 12 different methods of mitigation of gaseous emissions from livestock and manure storage. Application of manure additives can be a practical option in terms of application, logistics, and cost (Iowa State University Extension Outreach, 2020).

One of the types of manure additives is biochar, which is a solid carbonaceous by-product (char) obtained from pyrolysis, gasification, or torrefaction. It is a carbon-rich, porous, black material. The abundant sources of biochar can be sludge, food waste, agricultural and forestry residues, municipal and animal waste (Białowiec et al., 2018; Dudek et al., 2019; Pulka et al., 2019; Stepien et al., 2019; Swiechowski et al., 2019; Syguła et al., 2019; Wang et al., 2019). A recent review of biochar utilization in crop and livestock agriculture was presented elsewhere (Kalus et al., 2019). Characterizations of biochar such as surface area, porosity, hydrophobicity, pH, cation exchange capacity, and functional groups depend on feedstock and the temperature of treatment (Amin et al., 2016). Maurer et al. (2017) studied the effect of topically applied biochar that floated on swine manure for a month. Observation showed 12.7–22.6% reduction of NH_3_ emission, 12–30% for H_2_S, and 8.7–26% for indole. However, due to the complexity of the biochar, the mechanism of emission reduction still needs more investigation.

According to Zhu (2000), most of the malodor producing bacteria and H_2_S have pH in the range of 6.5–7.5. Raising the pH of manure by adding high alkaline biochar may cause a decrease in gaseous emissions, especially in VFAs that are considered as major malodor contributors. Mroz et al. (2000) state that decreasing manure pH may help to inhibit ammonium transformation into its volatile form of ammonia. On the other hand, a more basic pH near the manure-air interface can be helpful with mitigating H_2_S emissions from shallow pit-stored manure (Wi et al., 2019). It is also generally assumed that pH regulates microbial activity in manure, which in turn, is driving odorous compounds generation/utilization.

Most recently, we have explored the feasibility of using biochars properties to control the pH near the water-air interface in an idealized system where thin layers of biochars were applied to the clean water surface (Meiirkhanuly, 2019; Meiirkhanuly et al., 2019). The results proved that the surficial application of biochar to water was able to change both the pH near the water-air interface and the pH of the solution with time. The pH change was dependent on the biochar pH and water buffer capacity. The biochars had a different floatability as well. These results in Meiirkhanuly et al. (2019) warrant further research into the next logical step, i.e., testing the floatability of biochars on the surface of manures with different pH and other physicochemical properties. Furthermore, the impact of biochars on the manure-air interface pH can be further explored as a factor influencing gaseous emissions of odorous compounds that are sensitive to pH. Results could be used for further development of this technology to mitigate odorous emissions from other area sources such as wastewater treatment basins, landfills, lakes with nutrient imbalance.

The effect of topically applied biochar on spatial and temporal pH change of swine manure has never been studied. Taking into account that the pH of manure is crucial for generating emissions from manure, the study aims show if topically applied biochar can be used as a treatment in order to mitigate emission from swine manure in further studies. The research on manure pH modification due to biochar application could bring new knowledge for a better understanding of the mechanism of odor emission mitigation from manure by biochar floating covers, previously proven (Maurer et al., 2017).

We hypothesize that the application of biochar to manure as floating cover will increase the pH value; however, the degree of the influence depends on the depth, biochar alkalinity, initial manure pH, and manure buffering capacity.

**Figure 1:**
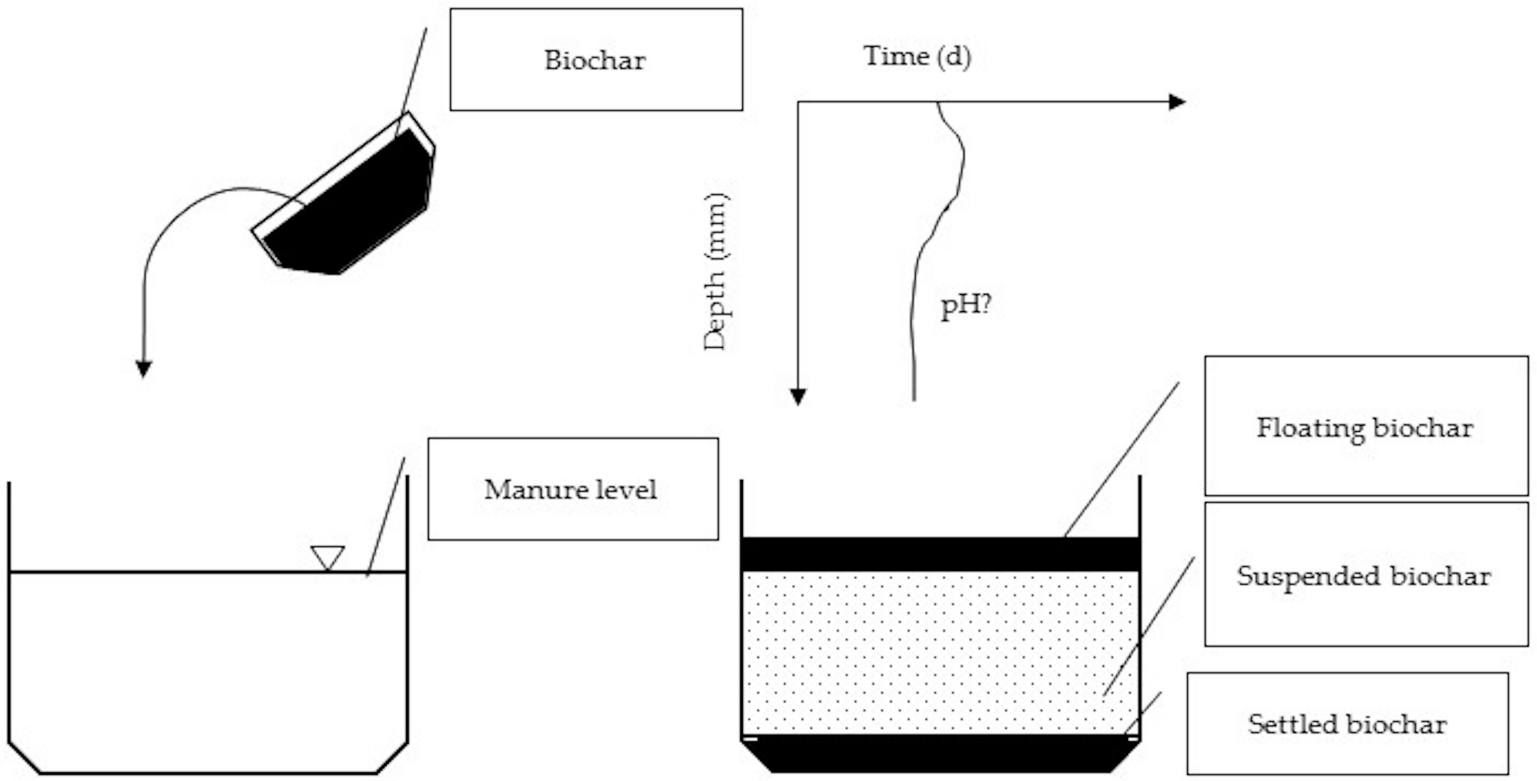
The ideation of the experiment on testing the spatial and temporal effects of topically applied biochar layer on manure pH.

The objective of this study is to test how highly alkaline and porous biochar (HAP) with pH = 9.20 and red oak biochar (RO) with pH = 7.50 (Table 1), applied on top of pit (pH = 8.00) and outdoor-stored (pH = 7.55) manure, can influence on spatial (every 1 mm) and temporal pH of manure, and by that, change their NH_3_ and H_2_S dissociation (Figure 1).

**Table 1:** Properties of the pit and outdoor-stored manure used in the experiment.

**Properties**	**Pit-stored**	**Outdoor-stored**
pH	8.00	7.55
Total solids (%)	4.07	2.60
Volatile matter (%)	71.01	66.54

## Materials and Methods

### Biochar

Description of methods of how properties of biochar were acquired is presented elsewhere (Meiirkhanuly, 2019). The detailed summary of the physicochemical properties of the two biochars HAP and RO was recently shown in Meiirkhanuly et al. (2019). Two different biomass residue corn stover and red oak wood biomass were used to prepare the HAP and RO, respectively. The particle size is <1 mm for both biochars. Briefly, the key differences were the pH and ash content of 9.2 vs. 7.5 and ~47 vs. ~16%, for HAP vs. RO biochars, respectively.

### Manure

Outdoor-stored manure was acquired from Crawford farm in North Central Iowa, and pit-stored manure was collected from Iowa Select Farms in Mid-West Iowa. Properties of manures that were used for the experiment are given in Table 1.

### The Determination of Biochar Type Influence on Spatial and Temporal pH Manure

Three of the food storage glass containers with a volume 1,700 mL (19 × 14.5 × 7.5 cm) were filled with 800 mL of pit manure each, and another three containers were filled with 800 mL of outdoor-stored manure. 6.35 mm thick layer of HAP and RO with 48 g and 58 g of mass, respectively, were applied on day 0, and pH measurement data were collected on days 0, 2, and 4. The matrix of the experiment is represented in Table 2.

**Table 2:** The matrix of the experiment.

**Treatments**	**Manure used**	
RO	Pit-stored	Outdoor-stored
HAP	Pit-stored	Outdoor-stored
Control (no biochar)	Pit-stored	Outdoor-stored

**Figure 2:**
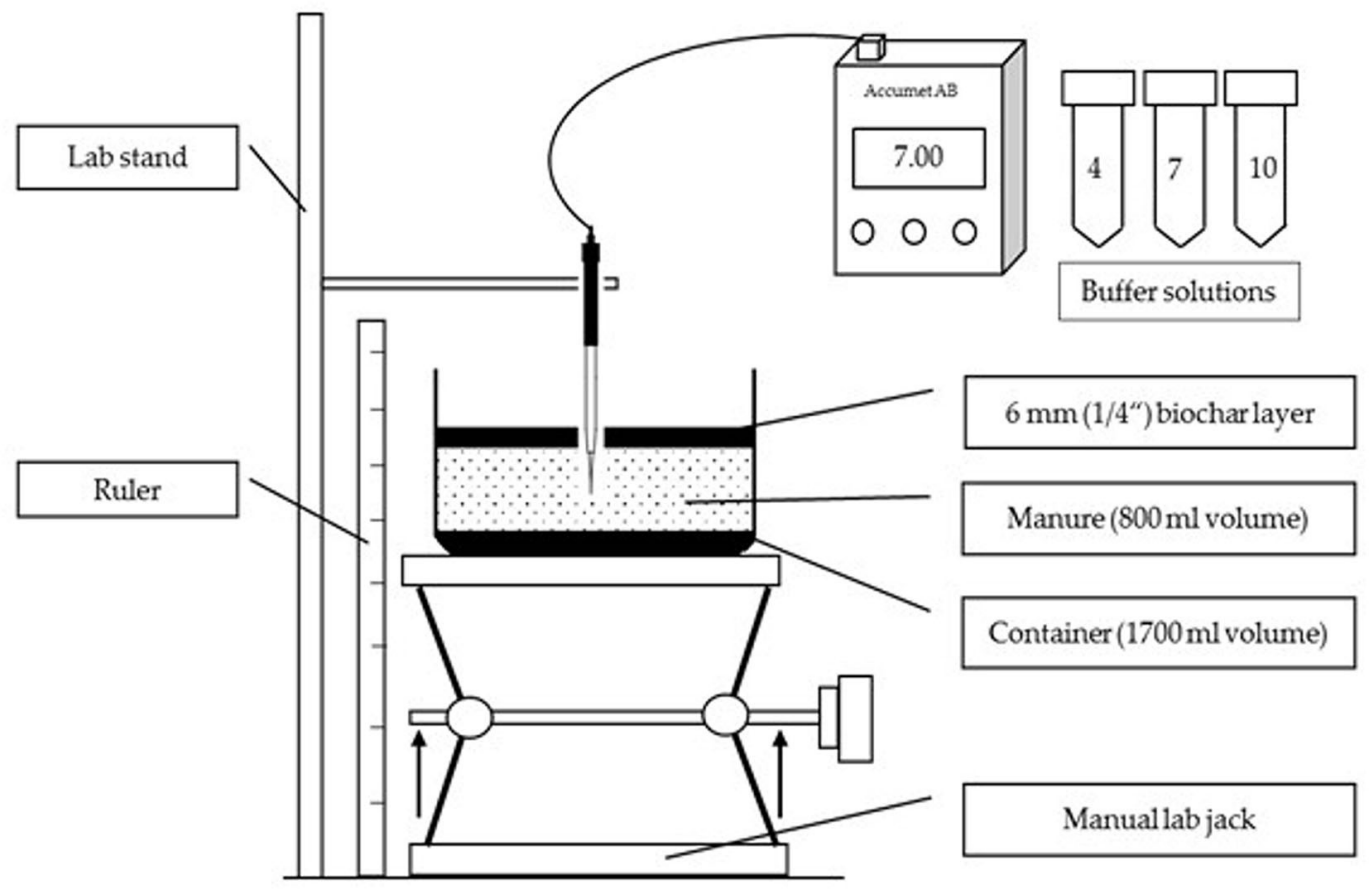
Experimental design for testing biochar influence on spatial and temporal pH distribution in swine manure.

Thin microelectrode (MI-415 Series Micro-Combination pH Probe, Microelectrodes, Inc., 2020), attached to a laboratory stand, was connected to an Accumet AB 15 pH meter (Fisher Scientific, 2020). A manual lab jack with a container of manure on the top of it was placed under the microelectrode (Figure 2). When the microelectrode penetrated the manure surface, pH measurements for every 1 mm of depth were collected by elevating the lab jack and using a ruler placed next to it.

### The Determination of Biochar Type Influence on Spatial and Temporal pH Manure

Buffer capacity of manure was determined by using the titration method. 1 M solution of acetic acid was made by adding 5.742 mL of stock solution to 100 mL of deionized water. After adding a drop of the solution, manure was stirred on a magnetic stirrer for 10 s and the pH of the manure was measured. Following equation was used to estimate the buffer capacity of manure:

$$\begin{array}{l} Buffer\;capacity=\;-\left(\frac{1}{slope}\right) \end{array}$$

Where the slope is fitted slope of the linear regression line for manure (Costello and Sullivan, 2014).

## Results

**Figure 3:**
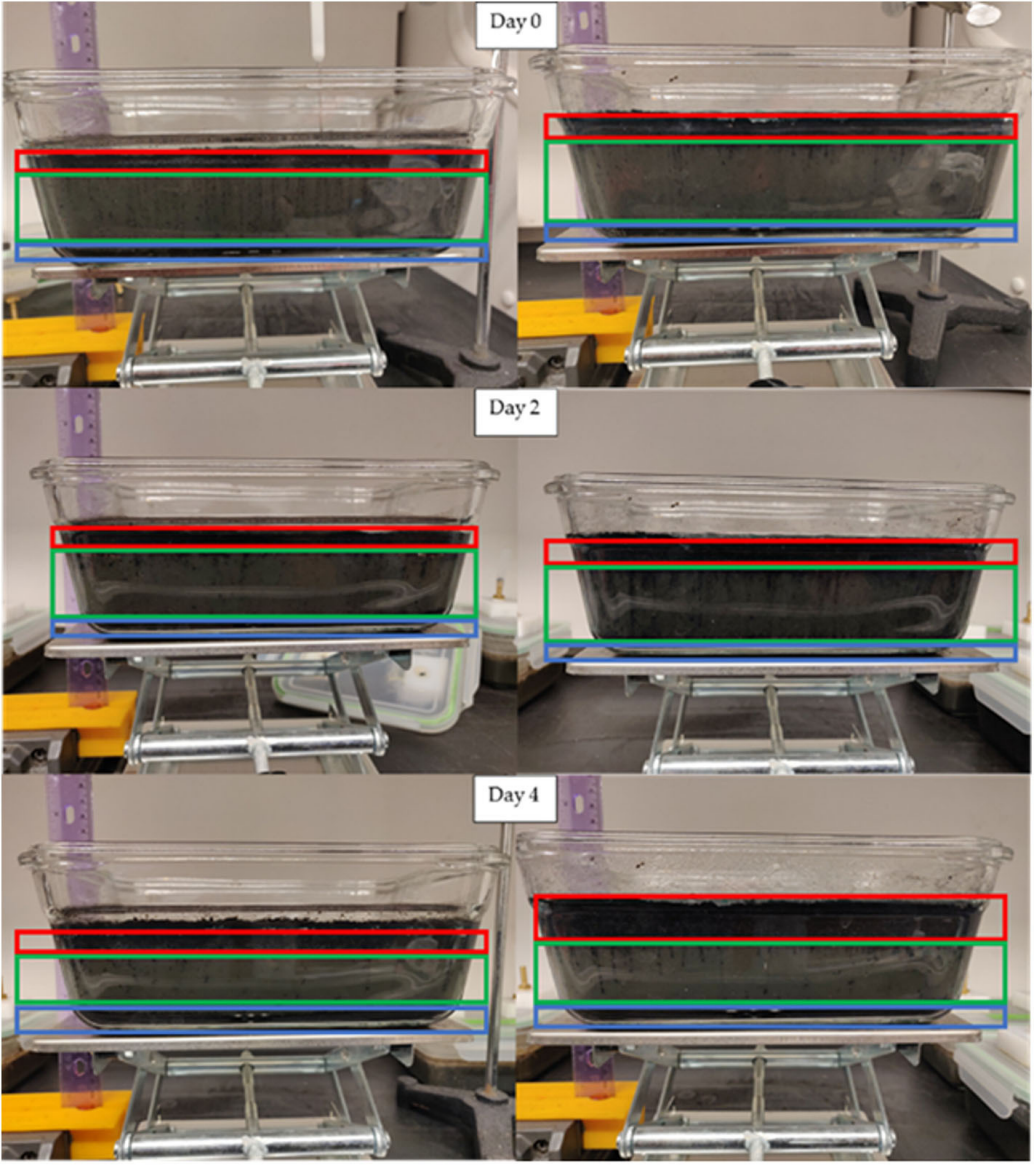
Photos of RO (left) and HAP (right) treated outdoor-stored manure on days 0, 2, and 4. Frames show biochar behavior (red—floating, green—suspended, blue—settled biochar).

**Figure 4:**
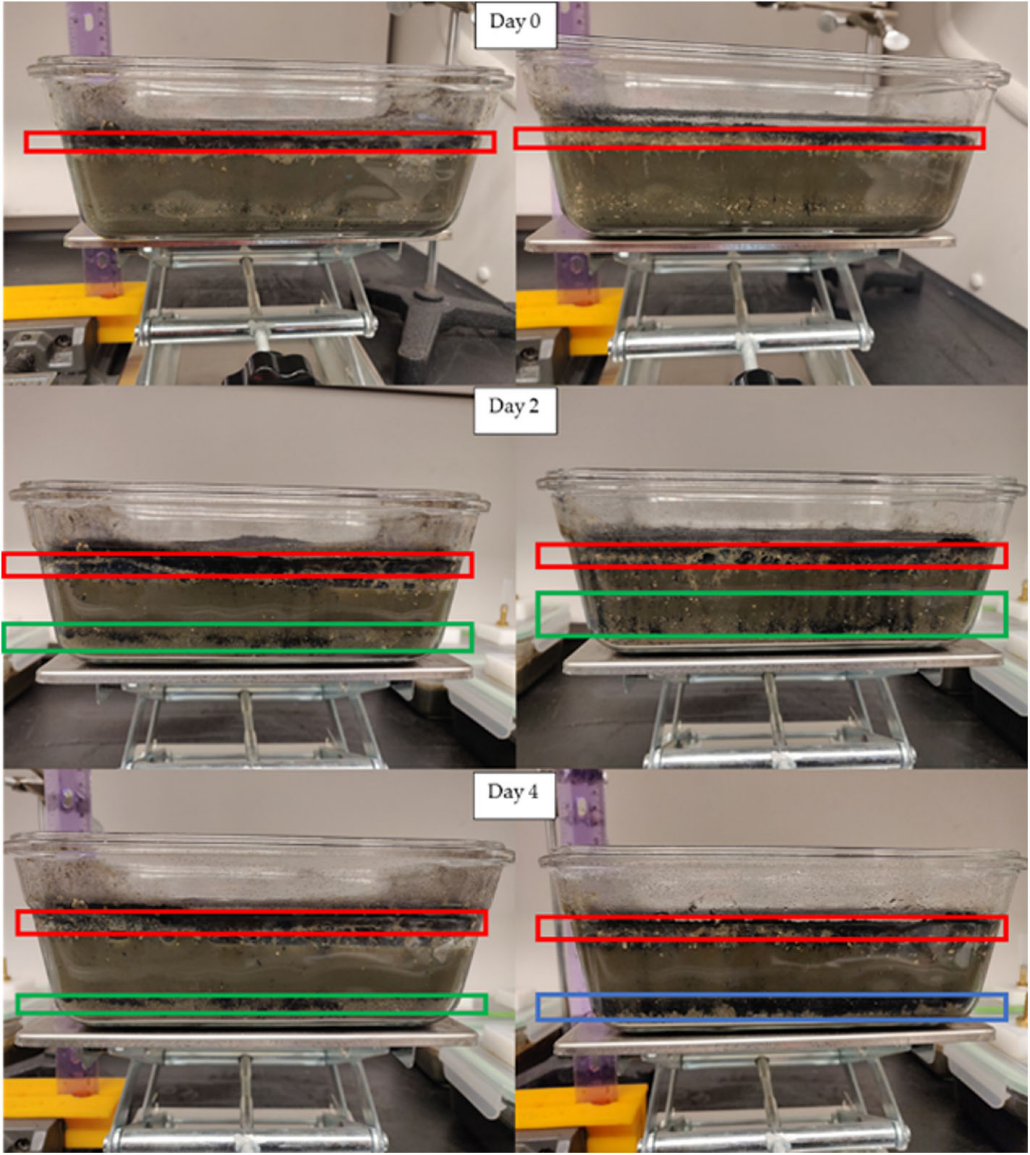
Photos of RO (left) and HAP (right) treated pit manure on days 0, 2, and 4. Frames show biochar behavior (red—floating, green—suspended, blue—settled biochar).

The biochars used in this study differ in characteristics as exhibited by the proximate analysis. The ash content of HAP is relatively high in comparison to RO, and SiO_2_ is the main component of the ash content in both biochars. After biochar application on day 0, both HAP and RO were floating on the surface of outdoor-stored manure. Biochars stayed on the top of the manure until day 4, and only a small fraction of biochars sank to the bottom. The HAP biochar had more suspended particles than RO (Figure 3). After biochar application on day 0, both HAP and RO were floating on the top of the pit manure. On day 2, the bottom of the HAP biochar layer crusted, and separation between the biochar layer and manure level occurred while RO biochar was floating on the top of the manure, and only a small fraction of it was suspended. On day 4, the separation between HAP biochar layer and manure became larger, and suspended particles of RO biochar settled on the bottom (Figure 4).

**Figure 5:**
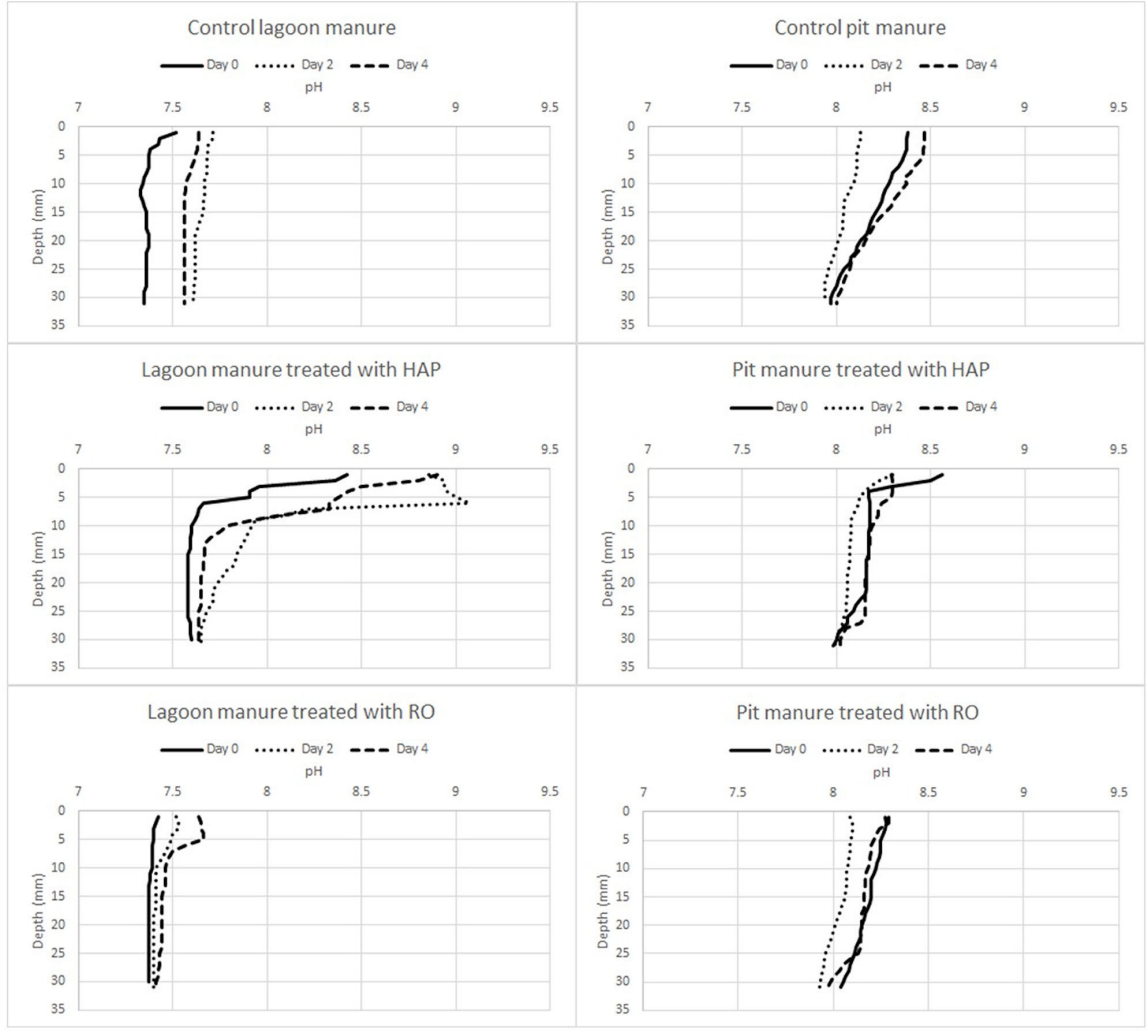
Spatial and temporal change pH in the outdoor-stored and pit-stored manure due to the influence of surficially applied biochar.

The summary of spatial and temporal change pH in the outdoor-stored and pit-stored manure due to the influence of surficially applied biochar is illustrated in Figure 5 and Supplementary Figure 1. On day 0, the pH range of outdoor-stored manure treated with RO (pH = 7.5) biochar was 7.42–7.37 (*p* = 0.960) from the surface to bottom whereas control manure had a pH range of 7.52–7.35. On day 2, control manure had pH 7.71–7.59, while RO changed the range of pH to 7.52–7.39 (*p* < 0.0001) from the surface to the bottom of manure. On day 4, control manure had a pH range of 7.64–7.57 and was similar to the pH of RO treated manure (7.64–7.39) (*p* = 0.033) from the surface of manure to the bottom of the container.

HAP biochar had consistently shifted the pH in the top 10 mm over the course of the experiment, especially for the outdoor-stored manure. On day 0 (40 min after application), HAP biochar increased outdoor-stored manure pH and ranged from 8.42 to 7.60 to (*p* < 0.0001), surface to the bottom, respectively. However, the greatest change in the pH due to HAP was in the top 10 mm (from 8.42 to 7.60), then it remained nearly the same to the bottom. Similarly, on day 2, the greatest change in the pH due to HAP was in the top 10 mm (from 8.86 to 7.92), with the maximum at ~6 mm depth. On day 4, manure surface pH was 8.9 and dropped to 7.79 at the 10 mm of depth and gradually changed to pH 7.66 at the bottom.

pH values of pit manure gradually dropped from the surface of manure to the bottom for all treatments. On day 0, the pH of the control manure on the surface was 8.38 and decreased to 7.90 on the bottom. HAP biochar changed manure pH from 8.56 at the surface to 7.91 on the bottom, with a sharp drop in the first 4 mm of depth. Then, below ~20 mm, it was within the range of the control. RO biochar changed the manure pH from 8.27 on the surface of manure, then started dropping up to 8.15 (*p* = 0.0.026) on 20 mm of depth.

On day 2, the pH of the control manure on the surface was 8.13 and decreased to 7.93 on the bottom. HAP biochar changed manure pH from 8.30 at the surface to 7.96 on the bottom, with a sharp drop in the first 5 mm of depth. The pH in the RO-treated manure was similar to control. On day 4, the control pH gradually decreased from the 8.47 at the surface to 7.99 at the bottom. The greatest pH changes for biochar-treated manure were observed in the top 10 mm of depth and again the nearest 10 mm to the bottom, the latter likely due to the biochar settling (Figure 4).

## Discussion

Outdoor-stored manure had the most apparent change in pH (~1) due to HAP biochar on day 0 (Figure 5) that continued to be even more noticeable (~1.5) on day 2 and 4 in the top 10 mm of depth (Supplemental Figure 1). On day 2, both biochars showed an apparent change of outdoor-stored manure where pH changed to 9.07 to 7.65 and 7.52–7.39 for HAP and RO, respectively, in comparison with pH of control manure pH 7.71–7.59. On day 4, the change of pH for HAP was still apparent in the top 10 mm of depth, where the drop was from 8.9 at the surface to 7.79 at 10 mm.

The changes in pH due to HAP treatment of pit-stored manure were not as noticeable as with the outdoor-stored manure. There was still an apparent change in pH in the top 5 to 10 mm near the surface (Supplemental Figure 1). Interestingly, the sharp drop in pH on day 0 at ~5 mm gradually moved to ~10 mm depth with biochar gradual settling on days 2 and 4 (Figures 3, 4).

**Figure 6:**
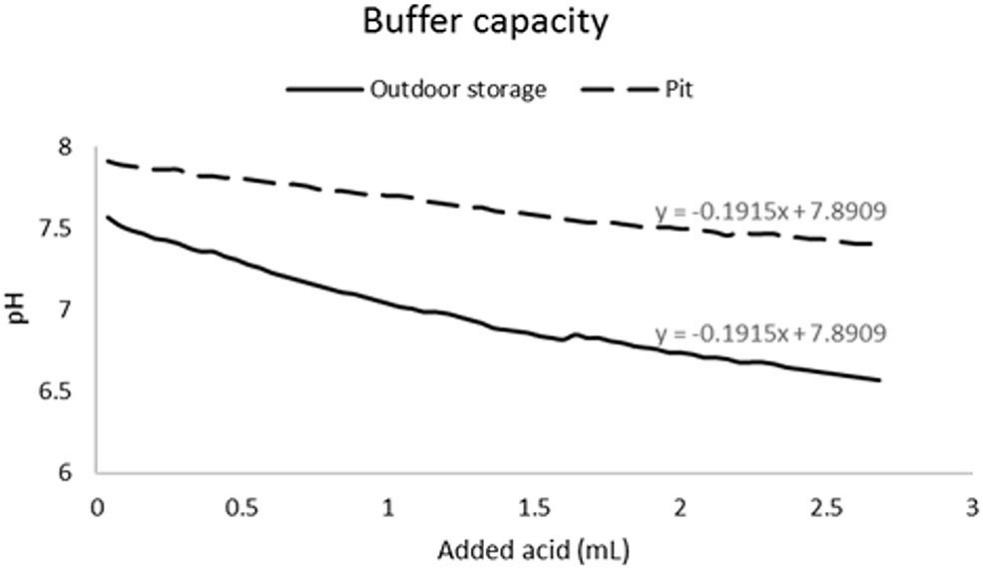
Buffer capacity of outdoor-stored and pit-stored swine manure estimated by the amount of acidity needed to drop the pH.

The pH changes due to RO treatment were less noticeable over time compared with those associated with the HAP treatment (Supplemental Figure 1). Outdoor-stored manure pH had noticeable change due to both biochars, especially for HAP. The pit manure had a smaller pH changes that were limited to the top ~5 mm on day 0 and 2 (Figure 5) for HAP and RO. Then, on day 4, the HAP treatment caused an ~0.8 shift in the pH in the top ~6 mm. Meiirkhanuly et al. (2019) shown that deionized water (with lower buffer capacity compared with tap water), had an immediate change in pH due to HAP and RO biochars. Similarly, to the controlled experiment with water, the reason for an apparent change in outdoor-stored manure pH in comparison with pit manure was due lower buffer capacity of outdoor-stored manure than pit manure (Figure 6).

Contrary to the experiment with water described in Meiirkhanuly et al. (2019), HAP biochar was floating much longer on top of outdoor-stored manure from day 0 to day 4. In the case of pit manure, the layer of HAP biochar bridged, and separation between the biochar layer and manure level occurred (Figure 4). RO biochar was floating on top of both outdoor-stored and pit manure (Figure 3).

**Figure 7:**
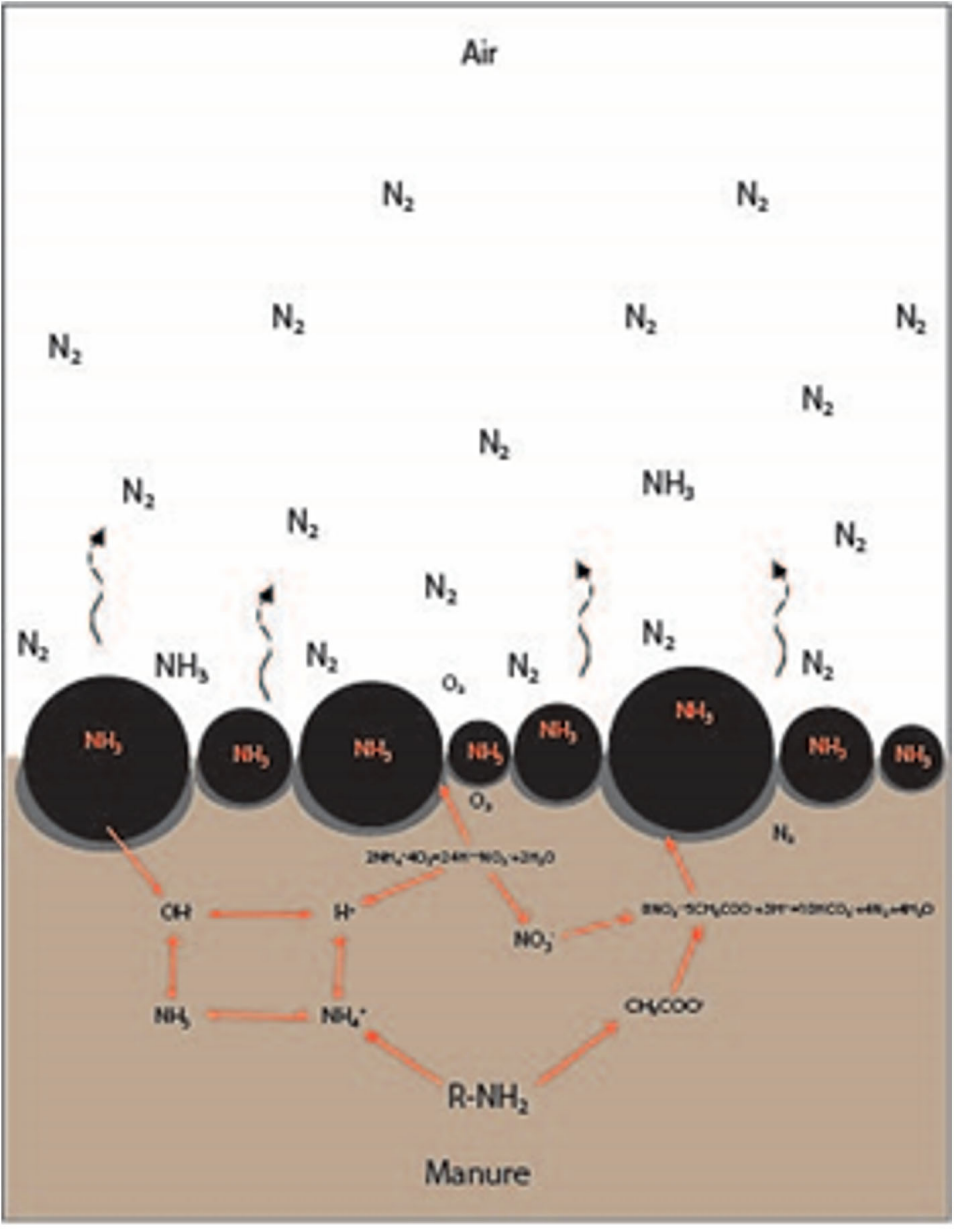
A preliminary model of the impact of biochar on the manure-air interface on the mitigation of NH_3_ emissions.

It is also important to hypothesize the mechanism of biochar interaction with NH_3_, a key air pollutant and mild odorant that is commonly considered for mitigation of gaseous emissions from area sources in animal agriculture. Figure 7 presents a model involving several chemical reactions that affect the pH at the manure-air interface. The source of ammonia nitrogen is the ammonification of N-organic compounds occurring in the deeper (anaerobic) zone of the manure storage pit. A fraction of the ammonia is adsorbed to biochar, a fraction is still dissolved in the manure, and some NH_3_ diffuses through the biofilm covering the surface of the biochar. At the same time, the O_2_ diffuses from the air-biochar-manure interface. It likely creates a microscale aerobic-anoxic-anaerobic gradient with decreasing redox potential. In the aerobic zone, O_2_ and ammonia are used for nitrification facilitated by nitrifiers present in the biofilm. Ammonia is then oxidized to nitrates. Next, products of ammonification and organic compounds degradation low weight fatty acids (acetate) are used for the denitrification (as a source of C). Denitrification in the anoxic zone leads to the transformation of nitrate to N_2_, which is then released to the air.

Biochar plays an important buffering role. Biochar releases the hydroxides, which neutralize H^+^ produced during nitrification. Biochar allows creating the expanded biofilm which is colonized by nitrifying and denitrifying bacteria responsible for the transformation of N compounds. Biochar facilitates creating the microenvironment where different processes of ammonification, nitrification, and denitrification occur. The next function of biochar is the adsorption activity, which bonds the load of ammonia nitrogen. Biochar also plays a role as a buffering agent of pH, which is important for maintaining suitable conditions for the growth of microorganisms. This mechanism still requires further investigation.

In this paper, we focused on the influence of two types of biochar on pH changes in manure, as a proof-of-the-concept of biochar application as a floating cover for odors emission mitigation. Our research showed that depending on biochar origin and properties, the expected effect may differ. Therefore, the next step in developing solutions should be more advanced research on the adsorption of odors by the biochar-manure system, specifically on the mechanisms potentially mitigating nuisance emissions. Also, the measurements of the concentration of pollutants adsorbed in biochar, for determining the mass balance of the pollutants' fate should be done. An important aspect should also be to observe the biological transformation of odors, especially ammonia and influence of OH^−^, and H^+^ transport on nitrification/denitrification (as we proposed the hypothesis by application of abduction reasoning—Figure 7). The next question to be addressed from the practical point of view is to determine the changes of the biochar density and floatability with time after application to manure.

The present research was a proof-of-the-concept, just an initial study for confirmation of the initial hypotheses; however, the mechanism of how biochar works as a buffering agent of pH still requires further discussion and explanations. The execution of Boehm's titration (Schönherr et al., 2018), FTIR, and XPS to properly evaluate the relationship between pH and biochar properties are crucial. Furthermore, the examination of the ash content influence by the determination of ions content in biochar and leaching should be done.

## Conclusion

Biochars applied to manure changed the spatial and temporal distribution of pH. Results showed that HAP biochar increased outdoor-stored manure pH, particularly within the top 10 mm of depth, where pH ranged from 8.90 to 7.79 on day 4. Both biochars increased pit manure pH in comparison with control on day 4. However, differences were not considerable. The reason for the insignificant effect of biochars on pit manure was likely due to its higher buffer capacity in comparison with the outdoor-stored manure.

## Data Availability Statement

All datasets generated for this study are included in the article/Supplementary Material.

## Author Contributions

AB, JK, and ZM designed the experiment. ZM performed the experiment. ZM, JK, AB, and CB analyzed the data and wrote the paper. JK contributed reagents, materials, and analysis tools. ZM, JK, and RB acquired funding. All authors contributed to the article and approved the submitted version.

## Conflict of Interest

The authors declare that the research was conducted in the absence of any commercial or financial relationships that could be construed as a potential conflict of interest.
